# Link Between Perception of Treatment Need and Craving Reports in Addiction

**DOI:** 10.3389/fpsyt.2021.790203

**Published:** 2022-01-31

**Authors:** Laura Lambert, Fuschia Serre, Berangere Thirioux, Nematollah Jaafari, Perrine Roux, Marie Jauffret-Roustide, Laurence Lalanne, Jean-Pierre Daulouède, Marc Auriacombe

**Affiliations:** ^1^University of Bordeaux, SANPSY, USR 3413, Bordeaux, France; ^2^CNRS, SANPSY, USR 3413, Bordeaux, France; ^3^Pôle Interétablissement d'Addictologie, CH Ch. Perrens and CHU de Bordeaux, Bordeaux, France; ^4^Unité de Recherche Clinique Intersectorielle en Psychiatrie à vocation régionale Pierre Deniker, Centre Hospitalier Henri Laborit, Poitiers, France; ^5^Université de Poitiers, Poitiers, France; ^6^Aix Marseille University, INSERM, IRD, SESSTIM, Sciences Economiques and Sociales de la Santé and Traitement de l'Information Médicale, Marseille, France; ^7^Cermes 3, Inserm U988, CNRS UMR 8236, Université de Paris, EHESS, Paris, France; ^8^British Columbia Centre on Substance Use, Vancouver, BC, Canada; ^9^Baldy Center for Law and Social Policy, Buffalo University of Social Sciences, New York, NY, United States; ^10^INSERM 1114, Department of Psychiatry and Addictology, University Hospital of Strasbourg, Fédération de Médecine Translationnelle de Strasbourg (FMTS), Strasbourg, France; ^11^Centre de Soins et d'Accompagnement et de Prévention en Addictologie (CSAPA), BIZIA, Médecins du Monde, Centre Hospitalier de la côte Basque, Bayonne, France; ^12^Department of Psychiatry, Perelman School of Medicine, University of Pennsylvania, Philadelphia, PA, United States

**Keywords:** perception of treatment need, clinical insight, substance use disorder, craving, addiction

## Abstract

**Background:**

Perception of treatment need (PTN), a component of clinical insight, is associated to negative addiction treatment outcomes when low. Our hypothesis was that lower PTN was associated with less craving when reported retrospectively, the most common measure of craving in clinical settings.

**Objective:**

To explore the association between PTN and craving among a dataset of subjects with severe substance use disorders.

**Methods:**

Participants were recruited from outpatient addiction clinic admissions or harm reduction program services. Good and low PTN were based on consistency between severe addiction (at least six DSM-5 criteria) and self-report need for addiction treatment from the Addiction Severity Index. Craving was retrospectively characterized over the past 30 days. Multiple regression analyses were conducted.

**Results:**

Participants with low PTN (*n* = 97) retrospectively reported less frequent and intense episodes of craving, compared with participants with good PTN (*n* = 566) after controlling for sociodemographic factors, addiction type, and severity (*p* < 0.0001).

**Conclusion:**

Low perception of treatment need among subjects with severe addictions is associated to less retrospective report of craving, which may contribute to reduced efficiency of treatment. Further studies are needed to explore the mechanisms of the association.

## Introduction

Addiction is characterized by an impaired control over use of reinforcing substances or behaviors and repeated relapses after attempts to stop (1, 2). Craving is reported as transient, intrusive, and unwanted experiences of urges for use of a substance or an addictive behavior (1, 3). Importantly, craving is a major risk factor for relapse (4, 5) and consequently considered a target for treatment (6, 7). In clinical practice, therapists usually evaluate craving by asking patients to self-report craving episodes that have occurred in the past days/weeks or since the previous interview. Accordingly, the patients' capacity to identify, remember, and self-report craving is paramount for the efficiency of therapies focused on craving management.

Despite the effectiveness and availability of treatments, only a minority of people initiate treatment for addiction [e.g., (8–10)]. Indeed, according to a global epidemiological survey of more than 70,000 people across all continents, only 39.1% of people with a current substance addiction (excluding tobacco) admit to needing medical care (11). Of these, only 7.1% actually received adequate treatment for their addiction (11). In addition to social and economic barriers, the belief that treatments are not effective (12), or cultural norms (13), there is another fundamental barrier to treatment, which is the lack of perceived need for treatment (11). Lack of perceived treatment need has been documented among adults with substance use disorders (SUD) (14). Perception of treatment need (PTN) is one of the three subdimensions of clinical insight according to the David model (15, 16). Clinical insight is the capability to recognize one's mental illness, to recognize its symptoms and consequences, to perceive need for treatment, and to consent to medical care [for review see (17, 18)]. PTN growth has been hypothesized as the last step of clinical insight growth after problem recognition and symptom identification and relabeling to illness (14).

Lower clinical insight level is often associated with a reduced capacity to recognize, estimate, and self-report symptoms or disorder severity [e.g., (19, 20)] and is associated to lower treatment response (17). It is possible that the capacity to recognize and/or self-report craving episodes may be impaired among people with a low clinical insight and low PTN. In usual clinical practice, if this was the case, such patients would be less likely to benefit from craving management treatments contributing to poorer treatment outcomes.

To date, only few studies have explored the association between some components of clinical insight and craving, and results are inconsistent. In one study, subjects with an “impaired awareness of drug-seeking behavior,” evaluated with task choice preference, reported more cocaine craving (21). In another study, no association was found between “perceived need to change one's own drug use,” also evaluated with task choice preference, and cocaine craving, but the sample was very small (*n* = 18) (22). In contrast, two studies reported lower or equal level of craving for alcohol among individuals with lower clinical insight evaluated on the self-reported Hanil Alcohol Insight Scale (23, 24). To our knowledge, no study has yet examined the association between PTN and craving.

As individuals with lower clinical insight level are known to underestimate their symptoms severity [e.g., (25)], we hypothesized that lower perception of addiction treatment need (PTN) will be associated to less craving when reported retrospectively. To test this, we examined the correlation between perception of treatment need and craving, self-reported retrospectively, among subjects with a severe addiction, and controlling for addiction and subject characteristics.

## Materials and Methods

### Cohort Description

Data were extracted from two cohort studies, the Addiction Aquitaine Cohort (ADDICTAQUI) between June 2012 and June 2019 (26), and the Bordeaux subsample of the COhort to identify Structural and INdividual factors associated with drug USe (COSINUS) between November 2016 and May 2018 (27).

ADDICTAQUI cohort participants were recruited from outpatient addiction treatment centers that met the *Diagnostic and Statistical Manual of Mental Disorders*, Fifth Edition (DSM-5) (1) criteria for a substance use disorder or behavioral addiction (e.g., pathological gaming) for at least one substance or behavior. Non-inclusion criteria were severe cognitive impairment or illiteracy.

COSINUS cohort participants were recruited from active substance users (illegal substances other than cannabis or diverted psychotropic medications) in harm reduction settings that had injected at least once during the month prior to inclusion. Non-inclusion criteria were to be under judicial protection.

Common inclusion criteria to the two samples were over 18-year-old, French-speaking, regular (2 times per week for the past 6 months) users of psychoactive substances and documented informed consent. All participants received a standard baseline clinical interview with a trained clinical research interviewer using the Addiction Severity Index (ASI), a numerical rating scale to assess craving, and the Mini International Neuropsychiatric Interview (MINI). Both cohorts were approved by French biomedical research regulations and ethical committees (CNIL, CPP, CEEI/IRB).

### Selection of Participants for This Study

Participants were selected for the current analyses if they met a current DSM-5 severe intensity diagnosis of a substance use disorder (≥6 criteria) or pathological gambling disorder (≥8 criteria) explored by the MINI.

Participants self-reported their main problematic use (main addiction) during the ASI interview [Drug and Alcohol section, Question 14 (D14)]. When more than one, or no main addiction was reported, the most severe was selected (highest number of criteria). Groups of main addiction with fewer than 40 subjects were excluded because such sample sizes were too small to control for the effects of multiple analyses.

**Figure 1:**
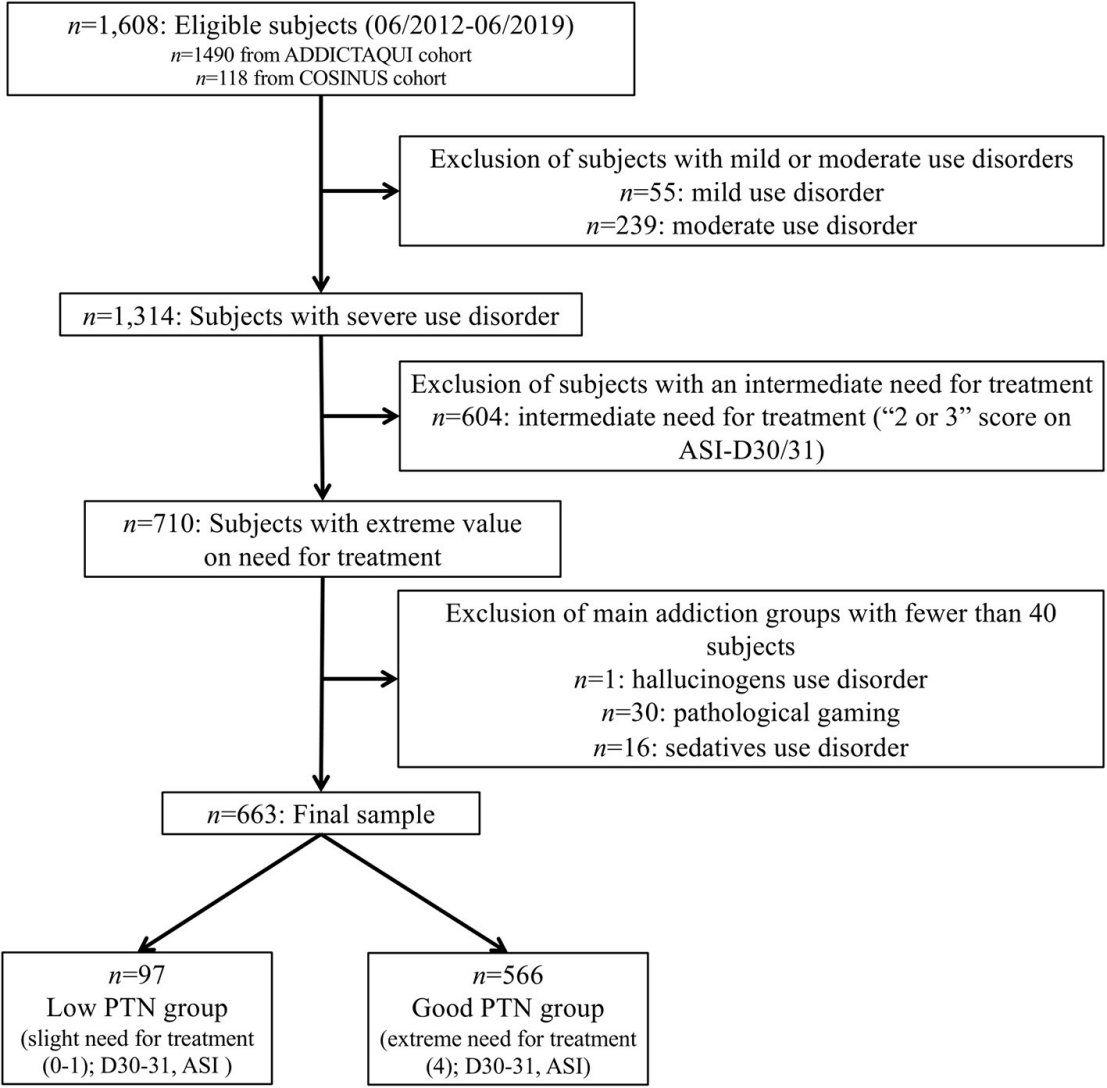
Flow chart of selection of subjects among ADDICTAQUI and COSINUS cohorts.

Thus, in total, 663 subjects were included in the study (Figure 1). This final sample was further divided into two groups according to the subjects' PTN status (see Section 2.3.4).

### Instruments and Measures

#### Mini International Neuropsychiatric Interview Adapted for DSM 5 Addiction

We adapted the MINI structured interview (28) to explore current DSM 5 addiction diagnosis (over the past 12 months) using the 9 criteria of gambling disorder and the 11 criteria of SUD (1, 2). More than one disorder was qualified as “*poly-addiction*.” “*Diagnostic severity*” was defined according to the standard DSM-5 cut-offs (mild: 2–3 criteria, moderate: 4–5 criteria, severe: ≥6 criteria) and “*Addiction criteria*” was defined according to the number of endorsed DSM-5 criteria.

#### Addiction Severity Index

The ASI is a semi-structured interview for a multidimensional assessment of problem substance users (29). We used a modified and validated French version of the ASI (m-ASI), adapted to include tobacco and behavioral addictions (30). The m-ASI explores use lifetime and over the past 30 days.

The ASI-D14 item determines the main problematic use according to the subject's opinion. After controlling for the DSM-5 diagnostic status, this substance/behavior was considered the “*main addiction*.”

The ASI-D30/31 items assess the subject's self-report “*Need for [main addiction] treatment”* using a 5-point scale: not at all (0), slightly (1), moderately (2), considerably (3), and extremely (4). For the purpose of this study, the variable “*Need for (main addiction) treatment”* was categorized: slight (scores: 0–1), mild (scores: 2–3), and extreme (score: 4), and only slight and extreme values (0–1 and 4) were considered.

“*Current use*” assesses the number of days of use in the past 30 days.

“*Duration of regular use*” assesses the number of years of regular use, which was defined as at least 2 times per week for 6 months or more.

#### Craving Evaluation

Craving was evaluated during a face-to-face interview through a self-report assessment covering the past 30 days. “*Craving frequency*” was defined as the number of days with craving (0–30). “*Mean intensity*” and “*maximal intensity*” ever experienced were collected using a numerical rating scale from 0 (no craving) to 10 (extreme craving). Craving was defined as “an intense desire and/or the occurrence of obsessive thoughts centered on the (*main addiction*).” This single-item method is the most used in clinical and research practices and is effective in assessing craving (6, 31, 32).

#### Perception of Treatment Need

For the purpose of this study, “*Perception of treatment need*” was modeled as the consistency between the objective interviewer measurement of “*Diagnostic severity*” (Severe Use Disorder according to DSM-5 cut-off MINI) and the subjective self-report evaluation of the “*need for (main addiction) treatment”* by participants (ASI-D30/31). This type of method, which contrasted objective (informant, clinician) vs. subjective (self-report, auto-questionnaire) measures, is often used to assess insight, including in addiction [e.g., (21, 22, 33–35)]. As shown in Figure 1, participants were considered having a “Good PTN” when they objectively presented with a severe disorder and subjectively self-reported an extreme need for treatment (ASI-D30/31 = 4). Subjects were considered having a “Low PTN” when they objectively presented with severe disorder but subjectively self-reported only slight need for treatment (ASI-D30/31 = 0 or 1). Analyses were conducted on two groups named “Low PTN” and “Good PTN,” with, respectively, 97 and 566 subjects.

#### Population

The variable “*Population*” was defined according to the cohort from which data were extracted: “*COSINUS”* or “*ADDICTAQUI.”*

### Statistical Analysis

Univariate analyses examined the association between craving variables (frequency, mean and maximal intensities) and PTN group, and with potential confounding factors: age, sex, bachelor's degree, addiction criteria, main addiction, past addiction treatment, current use (days), duration of regular use (years), and population. Additional analyses also compared PTN groups on sociodemographic variables and addiction-related factors, and examined the PTN–craving association in each main addiction groups separately (Supplemental Materials). Because of the non-normal distribution of variance, non-parametric tests were used for all univariate analyses according to variable types [Wilcoxon test (*z*), Kruskal–Wallis test (χ^2^), Spearman's correlation (ρ), or Pearson Khi-square test (χ^2^)].

Multiple generalized linear models (GLM) of Poisson tested the association between craving frequency and PTN and ordinal logistic regressions tested the association between craving intensities and PTN. All models used a backward stepwise selection and controlled for clinically relevant variables (age, sex, bachelor's degree, addiction criteria, population, main addiction), and variables moderately associated with craving in univariate analysis [*p* (enter) = 0.25; *p* (exit) = 0.1]. Inter-correlations between independent variables were tested to avoid multicollinearity [variance inflation factor (VIF) <5] before entrance in the same model. GLM produce coefficients that represent the predicted logarithm of counts of the dependent variable and the exponentiation of these coefficients places them on the same scale as the count variable [i.e., number of days of craving (craving frequency)] (36). The value of the exponentiated intercept (null model) represents a multiplicative change in the dependent variable for every one-unit change in the predictor (36).

Statistical analysis was performed using JMP Pro 15.0 (SAS Institute, Cary, North Carolina). The level of significance was set at *p* < 0.05. Bonferroni tests were applied to prevent the probability of a type I error. We conducted *n* = 54 univariate analyses, so the level of significance was set at *p* < 0.0009. Multiple generalized linear model and ordinal logistic regressions were FDR corrected.

## Results

### Sample Description

A total of 663 subjects were included in the analyses. The participants were primarily male (69%), with an average age of 38.9 (*SD* = 11.1) years, 49% had a bachelor degree, 14.6% (*n* = 97) had a low PTN, and 85.4% (*n* = 566) a good PTN. The most frequent main addiction was alcohol (43%), and a minority of subjects (17%) reported no craving episodes over the past 30 days. In ADDICTAQUI population (*n* = 586), 44 subjects (7.5%) had a low PTN and 542 (92.5%) had a good PTN. In COSINUS population (*n* = 77), 53 subjects (68.8%) had a low PTN and 24 (31.2%) had a good PTN (Supplementary Table 1).

### Univariate Analyses

**Figure 2:**
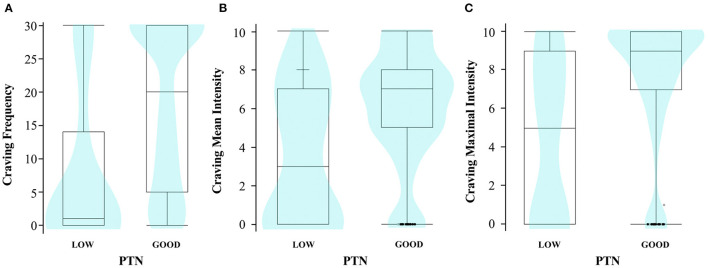
Diagram distribution of subjects according to PTN groups and craving parameters. (**A**) Frequency (*n* = 659); (**B**) mean intensity (*n* = 660); (**C**) maximal intensity (*n* = 662). The density diagram shows how the data are distributed for each craving value: the wider the diagram, the more subjects have that craving value (violin type, equal area size). The whisker boxes show the distributions of the data according to quartiles, range, and median; the isolated points are the outliers.

As described in Table 1 and Figure 2, subjects with a low PTN reported significantly less craving in terms of frequency (Wilcoxon test; *z* = −7.03; *r* = −0.27; *p* < 0.0001), mean intensity (Wilcoxon test; *z* = −5.02; *r* = −0.20; *p* < 0.0001), and maximal intensity (Wilcoxon test; *z* = −6.18; *r* = −0.24; *p* < 0.0001). Compared with those with a good PTN, all correlations had a small size effect. Craving was also slightly associated to a higher number of endorsed DSM-5 diagnostic criteria (Spearman tests; ρ = 0.14 for frequency; ρ = 0.21 for mean intensity; ρ = 0.23 for maximal intensity; *p* ≤ 0.002 for all) and was slightly less frequent and intense in COSINUS population (Wilcoxon tests; *z* = −3.73; *r* = −0.15 for frequency; *z* = −3.77; *r* = −0.15 for mean intensity; *z* = −3.47; *r* = −0.14 for maximal intensity; *p* ≤ 0.002 for all).

**Table 1:** Univariate analyses between craving parameters, PTN status, and confounding variables (demographic, addiction-related data).

	**Craving**								
	**Frequency**			**Mean intensity**			**Maximal intensity**		
	***n*/Mean (*SD*)**	**Coef**.	***P***	***n*/Mean (*SD*)**	**Coef**.	***P***	***n*/Mean (*SD*)**	**Coef**.	***P***
**Sociodemographic variables**									
Age_a_	*n =* 659	<0.01	0.931	*n =* 660	>-0.01	0.934	*n =* 662	<0.01	0.930
Sex_b_	*n =* 659	1.25	0.210	*n =* 660	2.00	0.046	*n =* 662	0.94	0.347
Male	16.9 (12.7)			5.6 (3.3)			7.2 (3.6)		
Female	18.2 (12.6)			6.1 (3.2)			7.5 (3.5)		
Bachelor's degree_b_	*n =* 655	0.60	0.548	*n =* 656	0.82	0.413	*n =* 658	<0.01	0.998
Yes	17.6 (12.4)			5.9 (3.0)			7.4 (3.4)		
No	17.1 (13.0)			5.6 (3.4)			7.1 (3.8)		
**Addiction-related factors**									
Addiction criteria_a_	*n =* 659	0.14	0.0002*	*n =* 660	0.21	<0.0001*	*n =* 662	0.23	<0.0001*
Current use (days)_a_	*n =* 640	0.29	<0.0001*	*n =* 639	0.13	0.003	*n =* 641	0.10	0.014
Duration of regular use (years)_a_	*n =* 643	0.15	0.0002*	*n =* 644	0.08	0.036	*n =* 646	0.09	0.027
Population_b_	*n =* 659	−3.73	0.0002*	*n =* 660	−3.77	0.0002*	*n =* 662	−3.47	0.0005*
ADDICTAQUI	18.0 (12.5)			5.9 (3.1)			7.5 (3.4)		
COSINUS	12.4 (13.2)			4.2 (3.8)			5.7 (4.3)		
Main addiction_c_	*n =* 659	24.97	<0.0001*	*n =* 660	12.55	0.014	*n =* 662	7.74	0.102
Alcohol	15.7 (12.4)			5.7 (3.3)			7.2 (3.7)		
Stimulants	19.1 (11.8)			6.3 (3.0)			8.0 (3.2)		
Cannabis	19.1 (12.1)			5.9 (2.5)			7.7 (2.8)		
Tobacco	21.4 (12.5)			6.4 (3.1)			7.7 (3.2)		
Opiates	14.6 (13.5)			4.8 (3.7)			6.2 (4.3)		
**Other factors**									
Current poly-addiction _b_	*n =* 659	0.25	0.803	*n =* 660	−0.71	0.479	*n =* 662	−1.46	0.144
Yes	17.3 (12.7)			5.6 (3.6)			7.3 (3.6)		
No	17.4 (12.8)			5.8 (3.3)			7.1 (3.6)		
Perception of treatment need (PTN)_b_	*n =* 659	−7.03	<0.0001*	*n =* 660	−5.02	<0.0001*	*n =* 662	−6.18	<0.0001*
Good	18.7 (12.2)			6.0 (3.0)			7.7 (3.3)		
Low	9.2 (12.3)			3.9 (3.7)			5.0 (4.3)		

Number of subjects (n) is mentioned in case of missing data for some subjects (full data n = 663). a. Spearman test (ρ); b. Wilcoxon test (z); c. Kruskal–Wallis test (χ^2^). Addiction-related factors are described only for the main addiction. Significant comparisons after Bonferroni corrections were marked (significance level: p <0.0009) using^*^.

Compared to those in the good PTN group (Supplementary Table 1), subjects in the low PTN group were less likely to be initiating treatment, more from COSINUS than from ADDICTAQUI population (Khi^2^ of Pearson; χ^2^ = 204.91; df = 1; *p* <0.0001) and more likely to have an opioid use disorder than an alcohol or tobacco use disorder (*post hoc* comparisons, *p* < 0.05). PTN groups did not significantly differ on sociodemographic variables or on other addiction-related factors (Supplementary Table 1). Significant small to moderate correlations were found between PTN and craving for alcohol (Wilcoxon test; *z* = −4.55; *r* = −0.27 for frequency; *z* = −4.73; *r* = −0.28 for maximal intensity; *p* < 0.0001 for all), opiates (Wilcoxon test; *z* = −3.44; *r* = −0.34 for frequency; *p* = 0.0006), and a tendency in the same direction for all other substances (Supplementary Table 2).

### Multiple Regression Models

Multiple regression analyses demonstrated that low PTN was significantly associated with less craving reports (*p* < 0.05 for the 3 parameters), after adjustment for sociodemographic, population, and addiction-related factors (Table 2). For craving frequency, subjects were predicted to have 17.29 days [*B* (intercept) = 2.85; e^2.85^ = 17.29] of craving when all other terms were zero. Thus, a subject with low PTN is expected to self-report, on average, 0.77 times fewer days of craving, i.e., 13.31 days less (17.29 × 0.77) than those with good PTN (*p* < 0.0001). A subject with low PTN is expected to self-report, on average, a mean intensity of craving 0.74 times lower and a maximal intensity 0.63 lower than those with good PTN (respectively: *p* < 0.05 and *p* < 0.001).

**Table 2:** Multiple models of predictors of craving frequency and craving mean and maximal intensities.

**Craving frequency**							
	***B***	***SE***	χ^2^ **Likelihood ratio**	***P*** **-value (FDRcor)**	**Exp (B)**	**Exp (** ***B*** **) 95% CI**	
						**Lower**	**Upper**
PTN (low vs. good)	−0.262	0.06	19.65	<0.0001*	0.770	−0.387	−0.143
Current use (days)	0.022	<0.01	55.82	<0.0001*	1.022	0.016	0.030
Addiction criteria	0.073	0.02	14.72	<0.001*	1.076	0.036	0.111
Main addiction (vs. opiates)			17.45	0.004*			
Alcohol	−0.133	0.05	7.60	0.006*	0.875	−0.228	−0.038
Tobacco	0.003	0.08	<0.01	0.970	1.003	−0.148	0.152
Cannabis	−0.040	0.06	0.42	0.518	0.961	−0.162	0.080
Stimulants	0.277	0.08	10.33	0.001*	1.319	0.110	0.440
Duration of regular use (years)	0.011	<0.01	6.60	0.018*	1.011	0.002	0.019
Age	−0.005	<0.01	1.71	0.287	0.995	−0.012	0.002
Sex (male vs. female)	−0.012	0.03	0.16	0.777	0.988	−0.073	0.049
Bachelor's degree (no vs. yes)	−0.006	0.03	0.04	0.835	0.994	−0.061	0.049
Population (COSINUS vs. ADDICTAQUI)	−0.040	0.06	0.42	0.661	0.961	−0.166	0.080
**Craving mean intensity**							
	***B***	***SE***	χ^2^ **of Wald**	***p*** **-value (FDRcor)**	**Exp (B)**	**Exp (** ***B*** **) 95% CI**	
						**Lower**	**Upper**
PTN (low vs. good)	−0.300	0.13	5.36	0.045*	0.741	−0.565	−0.038
Current use (days)	0.030	0.01	17.86	<0.001*	1.030	0.198	0.044
Addiction criteria	0.295	0.05	34.83	<0.0001*	1.343	0.198	0.393
Main addiction (vs. opiates)			14.25	0.018*			
Alcohol	−0.147	0.12	1.42	0.233	0.863	−0.393	0.098
Tobacco	0.233	0.21	1.29	0.257	1.262	−0.176	0.643
Cannabis	−0.315	0.16	3.65	0.056	0.730	−0.625	−0.005
Stimulants	0.564	0.22	6.38	0.012*	1.758	0.127	1.001
Duration of regular use (years)	0.020	0.01	3.39	0.097	1.020	−0.001	0.041
Age	−0.014	<0.01	2.09	0.189	0.986	−0.033	0.005
Sex (male vs. female)	−0.111	0.08	1.86	0.193	0.895	−0.270	0.048
Bachelor's degree (no vs. yes)	−0.026	0.07	0.12	0.725	0.974	−0.170	0.118
Population (COSINUS vs. ADDICTAQUI)	−0.399	0.15	6.97	0.025*	0.671	−0.707	−0.092
**Craving maximal intensity**							
	***B***	***SE***	*χ^2^* **of Wald**	***p*** **-value (FDRcor)**	**Exp (B)**	**Exp (** ***B*** **) 95% CI**	
						**Lower**	**Upper**
PTN (low vs. good)	−0.470	0.13	12.58	<0.001*	0.625	−0.730	−0.214
Current use (days)	0.027	<0.01	14.31	<0.001*	1.027	0.013	0.042
Addiction criteria	0.284	0.05	29.54	<0.0001*	1.328	0.181	0.387
Main addiction (vs. opiates)			7.18	0.201			
Alcohol	−0.078	0.13	0.36	0.550	0.925	−0.336	0.181
Tobacco	0.004	0.22	0.00	0.985	1.004	−0.418	0.043
Cannabis	−0.240	0.17	1.93	0.165	0.787	−0.569	0.090
Stimulants	0.543	0.24	5.17	0.023*	1.721	0.086	1.012
Duration of regular use (years)	0.024	0.11	4.51	0.074	1.024	0.002	0.046
Age	−0.012	0.01	1.43	0.337	0.988	−0.032	0.008
Sex (male vs. female)	−0.081	0.09	0.89	0.445	0.922	−0.249	0.087
Bachelor's degree (no vs. yes)	0.007	0.08	0.01	0.926	1.007	−0.145	0.159
Population (COSINUS vs. ADDICTAQUI)	−0.110	0.16	0.49	0.544	0.896	−0.417	0.200

Multiple regression models: generalized linear regression model of Poisson (frequency) and ordinal logistic regressions (intensities), fixed factors: age, sex, bachelor's degree, addiction criteria, main addiction, population. Statistics: Craving frequency: n = 620; R^2^ adjust. = 0.183; χ^2^ Likelihood ratio = 142.98; df = 12; log-likelihood (difference) = 71.49; AICc = 1082.94; overdispersion = 8.26; p (model) <0.0001; Pearson adjustment (χ^2^) = 5012.88; p (Pearson adjust.) = < 0.0001. Craving mean intensity: n = 620; R^2^ (U) = 0.04; χ^2^ = 97.58; df = 12; log-likelihood (difference) = 48.79; AICc = 2635.34; BIC = 2731.1; p < 0.0001. Craving maximal intensity: n = 622; R^2^ (U) = 0.04; χ^2^ = 85.84; df = 12; log-likelihood (difference) = 42.92; AICc = 2097; BIC = 2192.83; p < 0.0001. FDR, false discovery rate; B, estimate association parameter; Exp, exponential. Only p-values for substances are uncorrected. Significant comparisons were marked using^*^; significance level: p < 0.05. Main addiction: opiate group is used as reference for analyses.

Lower craving reports (all 3 parameters) were also found associated with fewer days of use over the past month (*p* < 0.001) and a lower number of DSM-5 criteria endorsed (*p* < 0.001) (Table 2).

## Discussion

Our main objective was to examine the association between perception of treatment need and craving, self-reported retrospectively, among subjects with severe addiction. PTN status was modeled as the consistency between objective addiction severity (severe use disorder according to DSM-5 cut-off) and the ASI self-report of the need for addiction treatment (slight or extreme) by participants. Adjusting for sociodemographic, addiction type, severity, and population, our results show, for the first time, that lower PTN was cross-sectionally associated with less frequent and intense episodes of craving self-reported retrospectively over the past month.

Our results are consistent with a previous study, which showed that lower clinical insight level was related to lower craving for alcohol self-reported retrospectively on the Obsessive-Compulsive Drinking Scale (OCDS) (23). Interestingly, some studies in addiction suggest that low clinical insight relates to earlier relapse (24, 37) or worse outcomes (e.g., drinking behavior) (38) after addiction treatment. Based on our results, we make the hypothesis that low clinical insight may negatively impact the ability to self-report craving retrospectively (the most common way craving is assessed in standard clinical practice) that in turn may interfere with participant's ability to seek treatment, and control craving when in treatment, and in turn be associated with a greater risk of relapse.

Although it is beyond the goal of this study to determine the underlying mechanisms of the association between PTN and craving, it is interesting to note that neuroimaging studies report that both are sustained by functional abnormalities in the same brain regions, i.e., anterior insula, anterior cingulate, and ventro-medial prefrontal cortices (vmPFC), that are involved in interoception (perception and processing of internal body signals) and in evaluating the personal relevance of stimuli (22, 25, 39).

Over-representation of subjects with AUD in our sample (43%) may have influenced the results as the toxicological features of alcohol are known to impair cognitive functions including memory (40, 41). However, subjects with AUD were mostly in the good PTN group compared with those with opioid use disorders (Supplementary Table 1). Moreover, correlation between PTN and craving was observed not only for alcohol (*p* < 0.001) but also for opiates (craving frequency; *p* < 0.001), and a trend in the same direction was also observed for all substances, except for tobacco craving mean intensity (Supplementary Table 2). It is also worth noting that the association persisted after adjustment for main addiction, past month consumption and duration of regular use, suggesting that it could be a common feature across addiction types and not just limited to the neurotoxic effects of alcohol.

Surprisingly, a large majority of our sample (85%) had a good PTN, whereas previous studies reported that individuals with addictions have mainly low clinical insight: 57% (42) and low PTN: 72% (14). One explanation is that our sample was mostly composed of subjects who self-initiated treatment (88% from ADDICTAQUI population), which may require a certain level of perception of treatment need. However, in our study, low PTN was also found in smaller proportions than in the literature in those not seeking treatment (from COSINUS cohort). Also, the overall good level of PTN in our sample could also be explained by the level of addiction severity, as only subjects with a “severe” use disorder were included in the analyses.

Contrary to our study, a study conducted among cocaine users showed that subjects with low “awareness of drug seeking behavior,” another sub-dimension of clinical insight, reported *more* craving (21). A possible reason to explain this result, in addition to the fact that this previous study was only among cocaine users and examined a different sub-dimension of clinical insight, may be the way craving was assessed. In our study, craving was assessed over a past period (retrospectively) and not currently (when it occurs), as in this other study. Indeed, low clinical insight in addiction has been previously linked to lower cognitive performances, especially executive functions, learning and episodic memories (43, 44), and could therefore be correlated with more difficulty to recollect past craving episodes.

The current study has several limitations that need to be acknowledged. First, our measurement of PTN was originally developed for the present study and is not yet validated. However, to assess PTN objectively and indirectly is less likely to be biased by social desirability compared with a direct (e.g., self-report scale) assessment (45, 46). Second, our sample was composed only of subjects with a severe addiction, thus results cannot be generalized to all individuals with addictions, especially those who have a less severe addiction. In the current study, we chose to exclude participants with mild to moderate addiction severity to contrast the objective severity and subjective report of PTN. Further studies should involve individuals with a wider range of addiction severity by evaluating the PTN differently. Third, we chose to compare only the extreme values for “Need for treatment” for which the differences should, in theory, be the most noticeable. However, we also tested the craving–PTN association with the full range of “Need for treatment” (0–4) evaluations and the results (data not shown) were consistent with those reported. Finally, PTN and craving were only evaluated once, cross-sectionally. Some theories suggest that clinical insight could fluctuate over time [e.g., (14, 33, 34)]. As craving is also a fluctuating phenomenon (47), it would be interesting to study how the fluctuations of one interact with the fluctuations of the other in daily life. Further studies should explore the effect of cognitive capacities on deficits in PTN or clinical insight and how they impact symptom perception and their retrospective report, and compare results when craving is assessed prospectively.

## Conclusion

This study explored the link between perception of treatment need, one of the three main sub-dimensions of clinical insight, and self-report of craving assessed retrospectively in a large sample of individuals with various addictions. Interestingly, based on a retrospective self-report, individuals with low perception of treatment need self-reported less frequent and intense episodes of craving, even after adjustment for addiction severity, and sociodemographic and population characteristics. This was not the case in individuals with a similar severity but a higher perception of treatment need. These results have important clinical implications when addiction treatment is focused on the management of craving. Future studies are needed to further investigate and better understand how PTN and more generally clinical insight impact craving, relapse, and treatment adherence.

## Data Availability Statement

The raw data supporting the conclusions of this article will be made available by the authors, without undue reservation.

## Ethics Statement

The studies involving human participants were reviewed and approved by University of Bordeaux. The patients/participants provided their written informed consent to participate in this study.

## Author Contributions

LLam, FS, and MA: conception, design of the study, writing—review, and editing the article. LLam: organized the database, performed the statistical analysis, and wrote the first draft of the article. MA, FS, LLam, and J-PD: data collection. MA, FS, NJ, BT, MJ-R, PR, and LLal: funding acquisition. All authors contributed to the article and approved the submitted version.

## Funding

This work was supported by Nouvelle-Aquitaine region (No. 2017-1R30114-00013238). Funding for COSINUS was supported by MILDECA Grant 2016. Funding for ADDICTAQUI was supported by Grant PHRC (2006- 2014) from the French Ministry of Health, Grant MILDT/MILDECA 2010, 2016 and office and staff support from Hospital Charles Perrens.

## Conflict of Interest

The authors declare that the research was conducted in the absence of any commercial or financial relationships that could be construed as a potential conflict of interest.

## Publisher's Note

All claims expressed in this article are solely those of the authors and do not necessarily represent those of their affiliated organizations, or those of the publisher, the editors and the reviewers. Any product that may be evaluated in this article, or claim that may be made by its manufacturer, is not guaranteed or endorsed by the publisher.
