# Supplementary material for: Link Between Perception of Treatment Need and Craving Reports in Addiction
Source: Front Psychiatry. 2022 Jan 31;12:790203. doi: 10.3389/fpsyt.2021.790203 (PMC8841420; doi:10.3389/fpsyt.2021.790203)
Supplement: Supplementary file 1 [file Data_Sheet_1.PDF]

Supplementary Materials

1 Factors associated with PTN group: univariate analyses

Statistical analysis

Low and good PTN groups were compared on socio-demographic variables (age, sex, bachelor's degree) and addiction related factors (addiction criteria, main addiction, current use, duration of regular use, current use problems, population, current poly-addiction), using Wilcoxon (z) and Khi^2 of Pearson tests ($\chi 2$), p-value set at 0.0009 after Bonferroni correction (Supplementary Table 1).

2 PTN – craving association by main addiction

Statistical analysis

Wilcoxon tests examined the association between craving variables (frequency, mean and maximal intensities) and PTN level (independent variable) for each main addiction, p-value set at 0.0009 after Bonferroni correction (Supplementary Table 2).

Supplementary Table 1. Demographic and substance-related characteristics in the Low PTN and Good PTN groups (*n*=663).

	Mean (*SD*) or percentage (*n*)		Coef.	*p*-value
	**Good PTN (*n*=566)**	**Low PTN (*n*=97)**		
Socio-demographic variables				
Age [a]	39.4 (11.1)	35.9 (10.7)	-3.17	0.002
Sex (male) [b]	67.5% (382)	77.3% (75)	3.74	0.053
Bachelor's degree (*n*=659, Yes) [b]	50.8% (286)	36.5% (35)	6.75	0.009
Addiction related factors				
Addiction criteria [a]	8.7 (1.6)	8.3 (1.7)	-2.21	0.027
Current use (days; *n*=642) [a]	21.2 (11.2)	19.8 (12.1)	-1.07	0.284
Duration of regular use (years; *n*=647) [a]	15.7 (10.9)	13.5 (10.1)	-1.78	0.076
Population [b]			204.91	**<.0001***
Addictaqui	95.8% (542)	45.4% (44)		
Cosinus	4.2% (24)	54.6% (53)		
Main addiction [b]			20.99	**<.001***
Alcohol	44.7% (253) [#]	30.9% (30)		
Cannabis	16.3% (92)	17.5% (17)		
Opiates	13.4% (76)	30.9% (30) [#]		
Stimulants	9.2% (52)	9.3% (9)		
Tobacco	16.4% (93) [#]	11.3% (11)		
Other factors				
Current poly-addiction (Yes) [b]	79.3% (449)	89.7% (87)	5.74	0.017

Notes: Number of subjects (*n*) is precise when data are missing when it is not *n*=663. Legends: a. mean (*SD*) Wilcoxon test (*z*); b. % (*n*) Khi^2 of Pearson; [#]: main addiction significantly different (post-hoc comparisons, $p < 0.05$). Addiction related factors are described only for the main addiction. Significant comparisons after Bonferroni corrections were marked using standard representation: *; significance level: $p < 0.0009$.

Supplementary Table 2. Univariates analyses between PTN and craving by main addiction.

	Craving								
	**Frequency**			**Mean intensity**			**Maximal intensity**		
	**Mean (*SD*)**	**Coef.**	***p*-value**	**Mean (*SD*)**	**Coef.**	***p*-value**	**Mean (*SD*)**	**Coef.**	***p*-value**
Alcohol	*n*=280	-4.55	**<.0001***	*n*=282	-3.25	*0.001*	*n*=283	-4.73	**<.0001***
Low PTN	6.7 (11.9)			3.3 (3.9)			4.0 (4.2)		
Good PTN	16.8 (12.1)			5.9 (3.2)			7.6 (3.5)		
Stimulants	*n*=61	-1.84	0.065	*n*=61	-2.20	*0.027*	*n*=61	-2.86	*0.004*
Low PTN	12.2 (12.2)			4.2 (3.0)			5.7 (3.7)		
Good PTN	20.3 (11.4)			6.7 (2.9)			8.4 (3.0)		
Tobacco	*n*=104	-2.40	*0.017*	*n*=103	-0.42	0.674	n=104	-1.91	0.056
Low PTN	12.1 (14.3)			5.5 (4.1)			6.0 (4.1)		
Good PTN	22.5 (11.8)			6.5 (2.9)			7.9 (3.0)		
Opiates	*n*=105	-3.44	**<.001***	*n*=105	-2.12	*0.034*	*n*=105	-1.74	0.082
Low PTN	7.6 (11.4)			3.4 (4.0)			4.6 (4.9)		
Good PTN	17.4 (13.4)			5.3 (3.5)			6.8 (3.9)		
Cannabis	*n*=109	-2.43	*0.015*	*n*=109	-2.36	*0.019*	*n*=109	-1.92	0.055
Low PTN	12.8 (13.1)			4.5 (2.9)			6.4 (3.5)		
Good PTN	20.2 (11.6)			6.1 (2.3)			8.0 (2.6)		

Notes: All analyses are Wilcoxon test (z). Significant comparisons after Bonferroni corrections were marked using standard representation: *; significance level: $p < 0.0009$.
